# Epigallocatechin-3-Gallate Alleviates High-Fat Diet-Induced Nonalcoholic Fatty Liver Disease via Inhibition of Apoptosis and Promotion of Autophagy through the ROS/MAPK Signaling Pathway

**DOI:** 10.1155/2021/5599997

**Published:** 2021-04-17

**Authors:** Dongdong Wu, Zhengguo Liu, Yizhen Wang, Qianqian Zhang, Jianmei Li, Peiyu Zhong, Zhongwen Xie, Ailing Ji, Yanzhang Li

**Affiliations:** ^1^Henan International Joint Laboratory for Nuclear Protein Regulation, School of Basic Medical Sciences, Henan University, Kaifeng, Henan 475004, China; ^2^School of Stomatology, Henan University, Kaifeng, Henan 475004, China; ^3^State Key Laboratory of Tea Plant Biology and Utilization, Anhui Agricultural University, Hefei, Anhui 230036, China

## Abstract

Nonalcoholic fatty liver disease (NAFLD) represents one of the most common chronic liver diseases in the world. It has been reported that epigallocatechin-3-gallate (EGCG) plays important biological and pharmacological roles in mammalian cells. Nevertheless, the mechanism underlying the beneficial effect of EGCG on the progression of NAFLD has not been fully elucidated. In the present study, the mechanisms of action of EGCG on the growth, apoptosis, and autophagy were examined using oleic acid- (OA-) treated liver cells and the high-fat diet- (HFD-) induced NAFLD mouse model. Administration of EGCG promoted the growth of OA-treated liver cells. EGCG could reduce mitochondrial-dependent apoptosis and increase autophagy possibly via the reactive oxygen species- (ROS-) mediated mitogen-activated protein kinase (MAPK) pathway in OA-treated liver cells. In line with *in vitro* findings, our *in vivo* study verified that treatment with EGCG attenuated HFD-induced NAFLD through reduction of apoptosis and promotion of autophagy. EGCG can alleviate HFD-induced NAFLD possibly by decreasing apoptosis and increasing autophagy via the ROS/MAPK pathway. EGCG may be a promising agent for the treatment of NAFLD.

## 1. Introduction

Tea, made from the leaves of *Camellia sinensis*, has long been considered a popular beverage worldwide [1–3]. Tea can be mainly classified into three types according to the manufacturing processes, including green tea (nonfermented), oolong tea (semifermented), and red and black teas (fermented) [4]. The functional constituents of tea can be attributable to the polyphenolic compounds, particularly catechins [1]. Four main catechins have been identified in green tea, such as epigallocatechin-3-gallate (EGCG), epigallocatechin, epicatechin-3-gallate, and epicatechin, with EGCG as the most active and abundant compound [3, 5]. These catechins have different hydroxyl groups on the B-ring with the presence/absence of a galloyl moiety [4]. EGCG exhibits strong binding to bioactive macromolecules, such as DNA and proteins via *π*-*π* stacking interaction, hydrogen bonding, and hydrophobic interaction [5, 6].

EGCG, a flavone-3-ol phenolic compound, has eight free hydroxyl groups [2], which might contribute to its diverse biological and pharmacological properties, such as antiamyloidogenic [7], chemopreventive [8], renoprotective [9], anticancer [10], antiaging [11], antiautoimmune [12], and antiviral [13] activities. Nonalcoholic fatty liver disease (NAFLD), a common chronic liver disease, has been considered one of the leading causes of end-stage liver disease, liver transplantation, and hepatocellular carcinoma [14–16]. The prevalence of NAFLD is growing in parallel with the global obesity epidemics, hypertension, type 2 diabetes, hyperlipidemia, and metabolic syndromes [15, 16]. It has been shown that EGCG could attenuate high-fat diet- (HFD-) induced NAFLD in rats and mice [17–19]. Nevertheless, the inhibitory effects and detailed mechanisms of EGCG in the initiation and progression of NAFLD need to be further investigated.

In this study, the mechanism of action of EGCG on the growth, apoptosis, and autophagy of oleic acid- (OA-) treated liver cells was elucidated. The HFD-induced NAFLD mouse model was further adopted to confirm the effect and mechanism of EGCG on NAFLD.

## 2. Materials and Methods

### 2.1. Cell Culture

Human liver cell lines L02 and QSG-7701 were purchased from Feiya Biological Technology Co., Ltd. (Yancheng, Jiangsu, China). The cells were cultured in Dulbecco's modified Eagle's medium (DMEM) with 10% fetal bovine serum (FBS) and 1% streptomycin/penicillin. Before each experiment, the cells were starved for 12 h in serum-free DMEM. Then, the cells were treated with the medium containing the 0.5 mM OA-bovine serum albumin (BSA; fatty acid-free, low endotoxin) complex (4 : 1, molar ratio), with or without a concentration of 50 *μ*M EGCG for 24 h. The medium with only BSA was selected as the control [20].

### 2.2. Oil Red O (ORO) Staining

Cells were fixed in 4% paraformaldehyde for 15 min, incubated with isopropyl alcohol for 20 min, and stained with ORO solution for 20 min, followed by being counterstained with hematoxylin at room temperature. The staining intensity of ORO was measured by ImageJ software (National Institutes of Health, Bethesda, MD, USA) [21].

### 2.3. Cell Growth Assay

The 5-ethynyl-2′-deoxyuridine (EdU) experiment was carried out using economical kits (RiboBio, Guangzhou, China). The cell proliferation rate was calculated as the percentage of positive cells to total cells. In addition, the cell counting kit-8 (CCK-8) detection kits (Beyotime, Shanghai, China) were used to detect cell viability. Cell viability was expressed as a percentage to the control group [22].

### 2.4. Flow Cytometry Assay

Cells were incubated with propidium iodide (PI)/RNase A mixture for 20 min. A FACSVerse flow cytometer (BD, San Jose, CA, USA) was adopted to analyze the cell cycle. The apoptotic level was examined by Annexin V-FITC/PI assay kits (KeyGen, Nanjing, Jiangsu, China) and further analyzed using a FACSVerse flow cytometer.

### 2.5. Immunofluorescence Staining

The green fluorescent protein- (GFP-)-red fluorescent protein- (RFP-) microtubule-associated protein 1 light chain 3 (MAP1LC3/LC3) plasmid has been used to detect the autophagic level [23]. Then, the GFP-RFP-LC3 plasmid (Hanbio, Shanghai, China) was transfected into the cells. After 48 h of incubation, the cellular fluorescence was determined using an Eclipse Ti fluorescent microscope (Nikon, Melville, NY, USA). The autophagosomes (yellow dots) and autolysosomes (red dots) were calculated as the ratios of positive-stained cells to total cells [24].

### 2.6. Monodansylcadaverine (MDC) Staining

Morphologically, the formation of autophagic vacuoles in the cytoplasm is a typical characteristic of autophagy. MDC is a key marker for autophagic vacuoles [25]. Briefly, the liver cells were stained with 50 *μ*mol/L MDC for 30 min at 37°C. Then, the cells were fixed with 5% paraformaldehyde and immediately observed under an Eclipse Ti fluorescent microscope (Nikon).

### 2.7. Measurement of Reactive Oxygen Species (ROS)

Cellular ROS levels were measured by 2′,7′-dichlorodihydrofluorescein diacetate (Beyotime).

### 2.8. Determination of Antioxidant Activity

The total superoxide dismutase (SOD) activity was determined using the kit with WST-8 (Beyotime). The catalase (CAT) activity was analyzed using the CAT assay kit (Beyotime). The glutathione peroxidase (GSH-Px) activity was detected by the GSH-Px assay kit with nicotinamide adenine dinucleotide phosphate (Beyotime).

### 2.9. Western Blot

Western blot assay was adopted to determine the expression levels of proteins. The primary antibodies, such as anti-cyclin D1/E1, anti-cyclin-dependent kinase (CDK) 2/4, anti-p21, anti-p27, anti-beclin-1, anti-P62, anti-LC3A/B, anti-extracellular signal-regulated protein kinase 1/2 (ERK1/2), anti-phospho- (p-) ERK1/2 (Thr202/Tyr204), anti-c-Jun N-terminal kinase (JNK), anti-p-JNK (Thr183/Tyr185), anti-p38, and anti-p-p38 (Thr180/Tyr182), and the horseradish peroxidase-conjugated secondary antibody were purchased from Cell Signaling Technology (CST, Danvers, MA, USA). Anti-B-cell lymphoma-2 (Bcl-2), anti-Bcl-2-associated X protein (Bax), anti-B-cell lymphoma-extra large (Bcl-xl), anti-Bcl-xl/Bcl-2-associated death promoter (Bad), anti-cleaved caspase-3/9, anti-cleaved poly-ADP-ribose polymerase (PARP), and anti-*β*-actin were obtained from Proteintech (Chicago, IL, USA). The bands were detected with a chemiluminescence system (Thermo, Rockford, IL, USA). Band intensities were analyzed by densitometry using ImageJ software.

### 2.10. Animals

The animal experiment was approved by the Committee of Medical Ethics and Welfare for Experimental Animals of Henan University School of Medicine (HUSOM-2017-208). C57BL/6J mice (8 weeks old, male), HFD (60% kcal as fat), and low-fat diet (LFD, 10% kcal as fat) were obtained from Vital River Laboratory Animal Technology Co., Ltd. (Beijing, China). All mice were maintained on a 12 h light/dark cycle and allowed access to food and water *ad libitum*. Mice were fed either HFD (*n* = 12) or LFD (*n* = 6) for a total of 14 weeks. After feeding for 10 weeks, HFD-fed mice were assigned to the HFD group (*n* = 6) and HFD+EGCG (50 mg/kg/day) group (*n* = 6). The mice were treated for an additional 4 weeks. Food/water intakes and body weights of the mice were measured. Then, the mice were killed and blood samples were collected. The liver, brown fat, and white fat were removed and weighed.

### 2.11. Biochemical Analysis

The concentrations of triglyceride (TG), total cholesterol (TC), alanine aminotransferase (ALT), and aspartate aminotransferase (AST) in the plasma were examined by an automated hematology analyzer (BC-6900, Mindray, Shenzhen, Guangdong, China). The contents of TG and TC in liver cells and tissues, as well as nonesterified fatty acid (NEFA) in liver tissues were detected by commercial enzyme-linked immunosorbent assay kits (Jiancheng Bioengineering Institute, Nanjing, Jiangsu, China).

### 2.12. Hematoxylin and Eosin (HE) Staining

Liver tissues were fixed in 10% neutral formalin, embedded in paraffin wax, sectioned at 4 *μ*m, and then stained with HE.

### 2.13. Immunohistochemistry (IHC)

Liver samples were respectively stained with anti-Ki67 (CST), anti-beclin-1, and anti-cleaved caspase-3 antibodies. The proliferation index, autophagic index, and apoptotic index were determined by the ratios of positive cells to total cells.

### 2.14. Statistics

All data were presented as the mean ± standard error of the mean. Differences between the two groups were determined by the two-tailed Student's *t*-test and one-way analysis of variance using GraphPad Prism 6 software. *P* < 0.05 was considered to indicate a statistically significant difference.

## 3. Results

### 3.1. EGCG Promotes the Growth of OA-Treated Liver Cells

**Figure 1:**
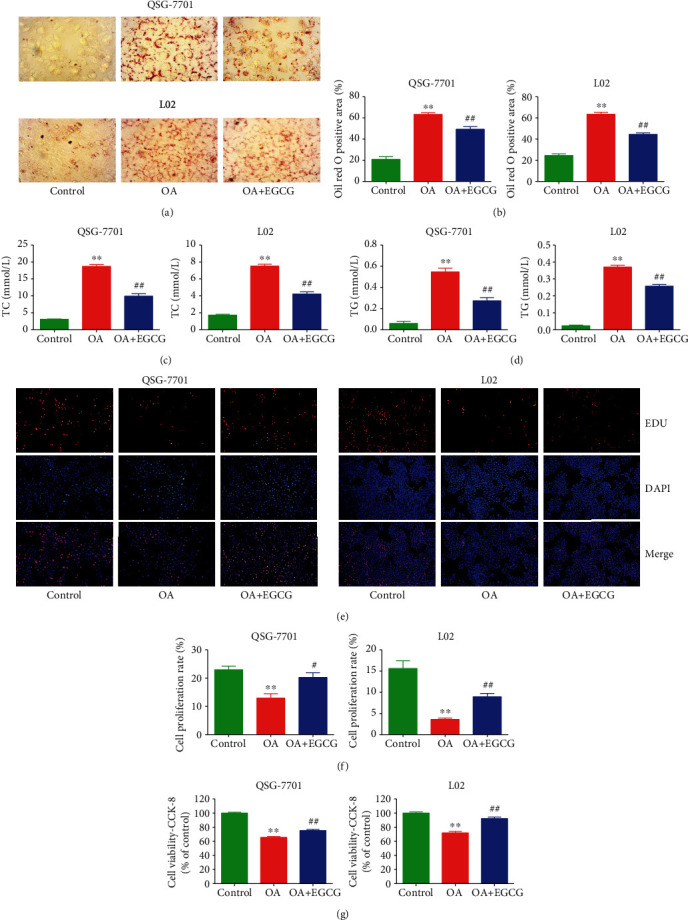
Effects of EGCG on lipid droplet formation and the growth of OA-treated QSG-7701 and L02 cells. (a) Representative photographs of ORO-stained QSG-7701 and L02 cells; original magnification ×400. (b) ORO-positive area was calculated. (c) The levels of TC were measured. (d) The levels of TG were measured. (e) DNA replication activities were examined by EdU assay; original magnification ×200. (f) The proliferation rate of each group was analyzed. (g) The percentages of viable cells were determined using CCK-8 assay, and the cell viability of control cells was normalized as 100%. Data are presented as mean ± SEM of three independent experiments; ^∗∗^*P* < 0.01 compared with the control group; ^#^*P* < 0.05, ^##^*P* < 0.01 compared with the OA group.

**Figure 2:**
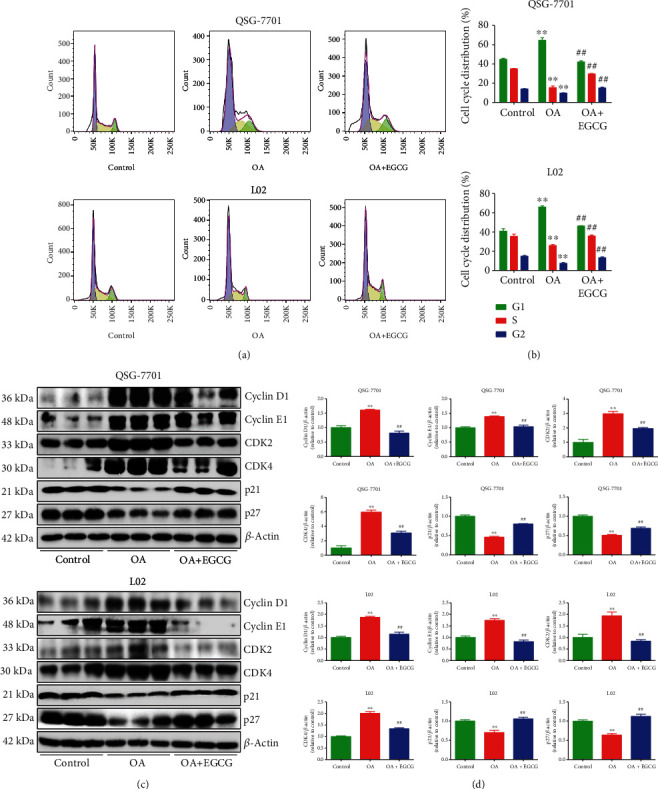
Effects of EGCG on cell cycle progression of OA-treated QSG-7701 and L02 cells. (a) Flow cytometry assay was used to determine cell cycle distribution. (b) Cell cycle distribution was analyzed. (c) Western blot analysis for the expression levels of cyclin D1, cyclin E1, CDK2, CDK4, p21, and p27 in each group. *β*-Actin was used as the loading control. (d) The densitometry analysis of each factor was performed in each group, normalized to the corresponding *β*-actin level. Data are presented as mean ± SEM of three independent experiments; ^∗∗^*P* < 0.01 compared with the control group; ^##^*P* < 0.01 compared with the OA group.

As shown in Figures 1(a) and 1(b), OA induced the accumulation of lipid in OA-treated liver cells, as further evidenced by the increased levels of TC and TG (Figures 1(c) and 1(d)). Treatment with EGCG reduced the lipid level in OA-treated liver cells (Figures 1(a)–1(d)). The viability and proliferation of liver cells were decreased by OA; nevertheless, EGCG promoted the viability and proliferation of OA-treated liver cells (Figures 1(e)–1(g)). In addition, the results showed that OA triggered cell cycle arrest at the G1 phase and EGCG reversed the trend (Figures 2(a) and 2(b)). Many cell cycle-related proteins have been identified in mammals, such as cell cycle regulatory proteins, including cyclin D1/E1 and CDK2/4, as well as inhibitory cell cycle regulators, including p21 and p27 [26, 27]. Our data suggested that OA increased the expressions of cyclin D1, cyclin E1, CDK2, and CDK4 but downregulated the protein levels of p21 and p27; however, administration of EGCG exhibited reverse trends (Figures 2(c) and 2(d)). Taken together, the data indicate that EGCG can promote the growth of OA-treated liver cells through promoting G1 phase cell cycle progression.

### 3.2. EGCG Decreases Apoptosis in OA-Treated Liver Cells

**Figure 3:**
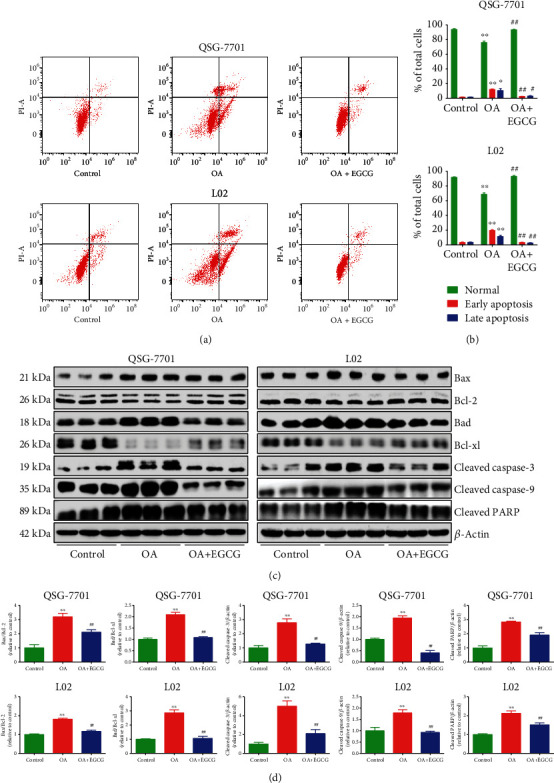
Effects of EGCG on the apoptosis of OA-treated QSG-7701 and L02 cells. (a) Flow cytometry assay was used to determine the apoptotic level. (b) The results of flow cytometry were analyzed. (c) Western blot analysis for the expression levels of Bax, Bcl-2, Bad, Bcl-xl, cleaved caspase-3/9, and cleaved PARP in each group. *β*-Actin was used as the loading control. (d) The densitometry analysis of each factor was performed in each group, normalized to the corresponding *β*-actin level. Data are presented as mean ± SEM of three independent experiments; ^∗^*P* < 0.05, ^∗∗^*P* < 0.01 compared with the control group; ^#^*P* < 0.05, ^##^*P* < 0.01 compared with the OA group.

As shown in Figures 3(a) and 3(b), the data suggested that OA increased the early and late apoptotic cell populations, whereas EGCG decreased the early and late apoptosis in OA-treated liver cells. The ratios of Bax/Bcl-2 and Bad/Bcl-xl are regarded as key factors in regulating apoptosis. Increased ratios of Bax/Bcl-2 and Bad/Bcl-xl are key phenomena in mitochondrial-dependent apoptosis in mammals [28, 29]. Furthermore, cleaved caspase-3/9 could induce apoptosis through the mitochondrial-mediated pathway [30]. PARP, a nuclear enzyme involved in DNA repair, is an important target for caspases during apoptosis [31]. The data showed that OA increased both the Bax/Bcl-2 and Bad/Bcl-xl ratios and the expression levels of cleaved caspase-3/9 and cleaved PARP, which were reversed by the administration of EGCG (Figures 3(c) and 3(d)). The results suggest that OA can induce mitochondrial-dependent apoptosis in liver cells and EGCG could reduce the apoptotic levels in OA-treated liver cells.

### 3.3. EGCG Increases Autophagy in OA-Treated Liver Cells

**Figure 4:**
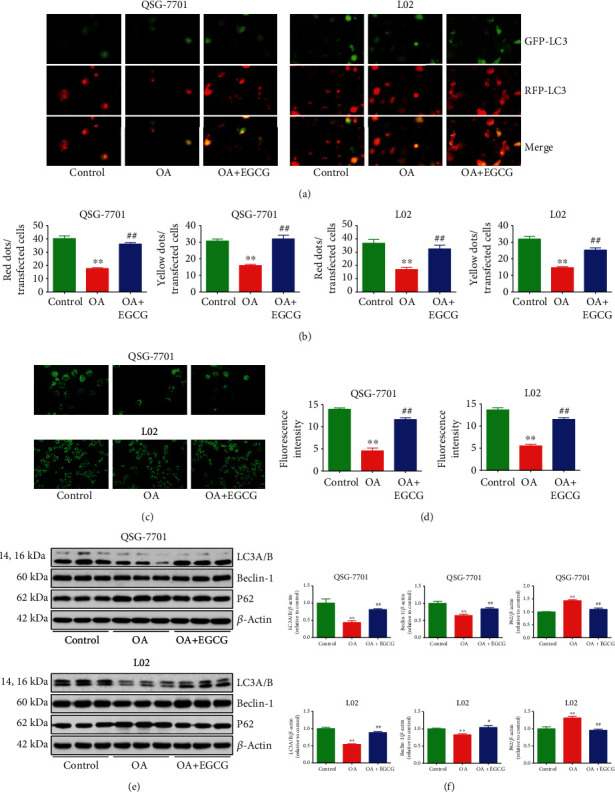
Effects of EGCG on the autophagy of OA-treated QSG-7701 and L02 cells. (a) GFP-RFP-LC3-transfected QSG-7701 and L02 cells were examined by fluorescence microscopy; original magnification ×1000. (b) The ratios of red and yellow dots to transfected cells were calculated. (c) Representative photographs of MDC staining. (d) The fluorescence intensity was analyzed. (e) Western blot analysis for the expression levels of LC3A/B, beclin-1, and P62 in each group. *β*-Actin was used as the loading control. (f) The densitometry analysis of each factor was performed in each group, normalized to the corresponding *β*-actin level. Data are presented as mean ± SEM of three independent experiments; ^∗∗^*P* < 0.01 compared with the control group; ^#^*P* < 0.05, ^##^*P* < 0.01 compared with the OA group.

Autophagy is responsible for the degradation of intracellular protein aggregates, invasive pathogens, and damaged organelles and therefore is essential in maintaining cellular homeostasis and responding to stress conditions [32, 33]. A crucial step in autophagy is the conversion of LC3 from the nonlipidated form (LC3-I) to the lipid-conjugated form (LC3-II) [33, 34]. Autophagic turnover could be molecularly monitored using a GFP-conjugated LC3 and the conversion of LC3-I to LC3-II [35]. In the present study, the GFP-RFP-LC3 plasmid was transfected into liver cells and further detected by fluorescence microscopy. Treatment with OA decreased the numbers of free red dots (autolysosomes) and yellow dots (autophagosomes), whereas administration of EGCG showed the opposite effects (Figures 4(a) and 4(b)). A similar trend was observed in MDC staining (Figures 4(c) and 4(d)). Apart from LC3, beclin-1 and P62 have also been considered specific markers of autophagy [33, 36]. The expression levels of beclin-1 and LC3 in the OA group were lower than those in the control group, but the protein levels of these two factors were higher in the OA+EGCG group than in the OA group. Furthermore, the expression level of P62 exhibited a reverse trend (Figures 4(e) and 4(f)). These results together suggest that the autophagic level is downregulated in OA-treated liver cells and treatment with EGCG could upregulate the autophagy machinery.

### 3.4. EGCG Suppresses the ROS/Mitogen-Activated Protein Kinase (MAPK) Pathway in OA-Treated Liver Cells

**Figure 5:**
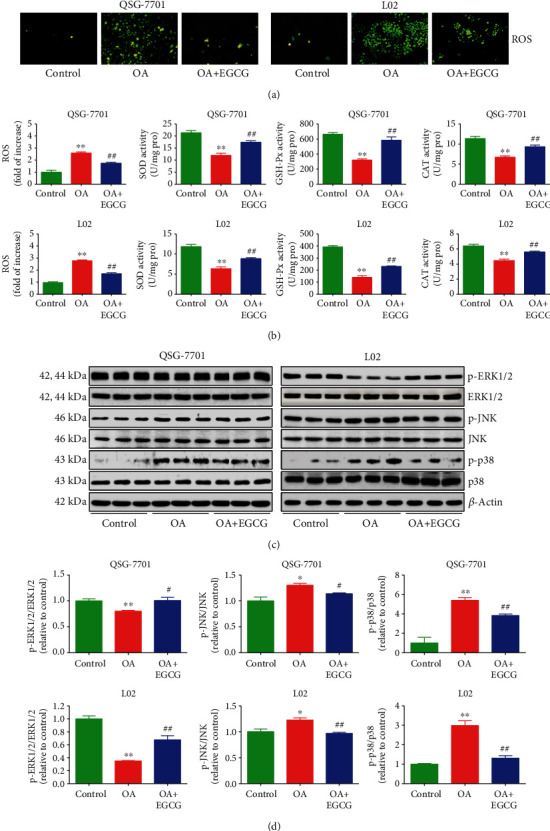
Effects of EGCG on the ROS/MAPK signaling pathway in OA-treated QSG-7701 and L02 cells. (a) The intracellular ROS production was detected using the fluorescent probe DCF-DA (shown in green; original magnification, ×400). (b) The intracellular ROS production and the activities of SOD, GSH-Px, and CAT were measured. (c) The protein expressions of ERK1/2, p-ERK1/2, JNK, p-JNK, p38, and p-p38 were analyzed by Western blot. *β*-Actin was used as the loading control. (d) The densitometry analysis of each factor was performed in each group, normalized to the corresponding *β*-actin level. Data are presented as mean ± SEM of three independent experiments; ^∗^*P* < 0.05, ^∗∗^*P* < 0.01 compared with the control group; ^#^*P* < 0.05, ^##^*P* < 0.01 compared with the OA group.

ROS such as hydroxyl radical, hydrogen peroxide, and superoxide anion are normally generated as by-products of aerobic metabolism [37, 38]. ROS can be scavenged by the antioxidant defense system that mainly includes GSH-Px, SOD, and CAT [37–39]. Compared with the control group, the ROS levels were increased, but GSH-Px, SOD, and CAT activities were downregulated in the OA group, which can be reversed by the administration of EGCG (Figures 5(a) and 5(b)). The results suggest that EGCG can reduce OA-induced oxidative stress in liver cells. It has been shown that ROS can activate the MAPK pathway and attenuation of ROS by ROS scavengers could deactivate MAPK signaling [40, 41]. As shown in Figures 5(c) and 5(d), OA reduced the expression of p-ERK1/2 but increased the levels of p-JNK and p-p38, while administration of EGCG exhibited reverse effects on the kinases. The data suggest that EGCG may suppress the ROS/MAPK pathway in OA-treated liver cells.

### 3.5. EGCG Attenuates HFD-Induced NAFLD in Mice

**Figure 6:**
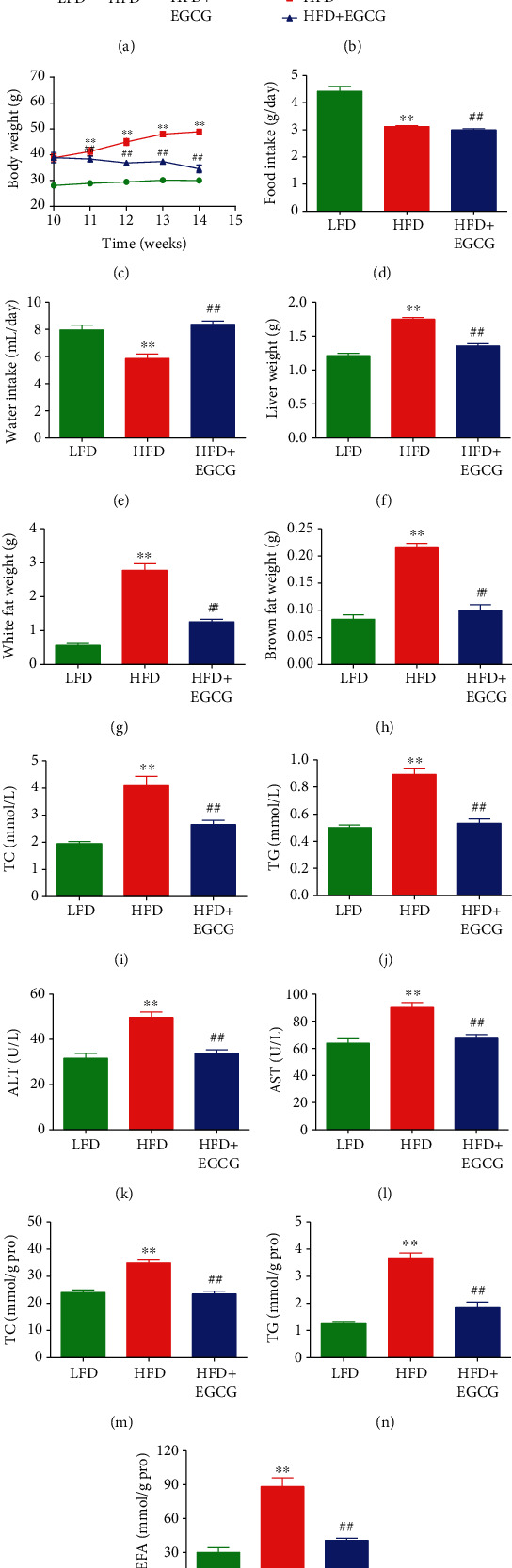
Effects of EGCG on HFD-induced NAFLD in mice. (a) Representative photographs of mice in each group. (b, c) The body weights of mice were measured. (d, e) Food intake and water intake were determined. (f–h) The liver weight, white fat weight, and brown fat weight were calculated. (i–l) The levels of TC, TG, ALT, and AST in the plasma of mice were detected. (m–o) The expression levels of TC, TG, and NEFA in the liver of mice were detected. Data are presented as mean ± SEM (*n* = 6). ^∗^*P* < 0.05, ^∗∗^*P* < 0.01 compared with the control group; ^##^*P* < 0.01 compared with the OA group.

**Figure 7:**
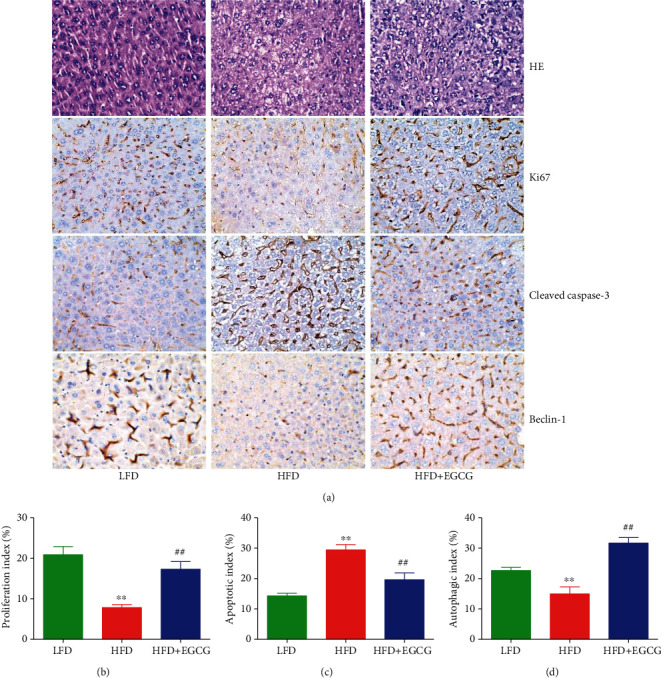
Effects of EGCG on the proliferation, apoptosis, and autophagy in liver tissues of the NAFLD mice. (a) Representative photographs of HE, Ki67, cleaved caspase-3, and beclin-1 staining in the liver of mice; original magnification ×400. (b–d) The proliferation index, apoptotic index, and autophagic index were calculated. Data are presented as mean ± SEM (*n* = 3). ^∗∗^*P* < 0.01 compared with the control group; ^##^*P* < 0.01 compared with the OA group.

Compared with the mice fed with LFD for 10 weeks, HFD-fed mice exhibited increased body weight, indicating that an alimentary obesity model had been successfully established. In addition, compared to the LFD group, HFD-fed mice showed decreased food and water intakes, as well as increased weights of liver, white fat, and brown fat. Treatment with EGCG reversed these changes except for the food intake (Figures 6(a)–6(h)). Furthermore, HFD-fed mice exhibited increased levels of TC, TG, ALT, and AST when compared to the LFD group, which could be reversed by the treatment with EGCG (Figures 6(i)–6(l)). Moreover, HFD-fed mice showed upregulated levels of TC, TG, and NEFA in the liver when compared with the LFD group, which were reduced by the administration of EGCG (Figures 6(m)–6(o)). Compared to the LFD group, the HFD group exhibited a higher apoptotic index, as well as a lower proliferation index and autophagic index, which could be reversed by the administration of EGCG (Figures 7(a)–7(d)). These results indicate that EGCG can attenuate HFD-induced NAFLD in mice.

## 4. Discussion

NAFLD, the most common chronic liver disease, leads to end-stage liver disease, liver transplantation, and hepatocellular carcinoma [14–16]. It has been shown that EGCG plays important biological and pharmacological roles in mammals. Nevertheless, the effect and mechanism of EGCG in the process of NAFLD are largely unknown. Human normal liver cells QSG-7701 and L02 have been widely adopted to investigate the mechanism of action of novel drugs and donors [42, 43]. OA, a monounsaturated fatty acid, has been successfully used in the establishment of the NAFLD model [44]. In this study, QSG-7701 and L02 cells were adopted to examine the effects of EGCG on NAFLD induced by OA *in vitro*. A recent study has revealed that epoxy stearic acid, a type of oxidative product from OA, can induce cytotoxicity and G0/G1 phase cell cycle arrest in HepG2 cells [45]. Our data indicated that OA decreased the viability and proliferation of liver cells and induced G1 phase cell cycle arrest. The changes could be reversed by the administration of EGCG. These data together indicate that EGCG acts as an effector molecule in promoting the growth of OA-treated liver cells.

Apoptosis is a conserved cell death pathway which can play key roles in the maintenance of organismal homeostasis and normal eukaryotic development [46, 47]. Two main apoptotic pathways have been identified in mammals: the mitochondrial-mediated intrinsic pathway and the death receptor-mediated extrinsic pathway [48]. Bcl-2 family proteins are involved in the regulation of apoptosis, such as Bax, Bad, Bcl-2, and Bcl-xl [49]. Many apoptotic stimuli can activate caspases, and PARP is activated by cleaved caspase-3, leading to the occurrence of apoptosis [31, 38, 49]. It has been reported that OA could induce apoptosis by increasing the levels of Bax and PARP but decreasing the level of Bcl-2 in HepG2 cells [50]. Another study shows that OA can promote the expressions of both cleaved caspase-3 and PARP1 [51]. Similarly, our data indicated that OA induced the early and late apoptosis, as well as increased the ratios of both Bax/Bcl-2 and Bad/Bcl-xl and the expressions of cleaved caspase-3/9 and cleaved PARP in liver cells. EGCG significantly reduced the apoptotic levels in the OA group. The data suggest that the apoptotic levels are increased in OA-treated liver cells and treatment with EGCG could reduce apoptosis.

Autophagy, an evolutionarily conserved catabolic pathway, serves to deliver cytoplasmic materials to lysosomes for recycling and degradation, leading to macromolecular synthesis and energy production [36, 52]. Autophagy is activated by many environmental factors, including cytokines, hormones, and nutrients [53]. Recent studies have indicated that autophagy is impaired in lipid-overloaded hepatocytes and in the liver from the NAFLD murine model and NAFLD patients [54–56]. In line with the above studies, we observed that the autophagic levels were decreased in OA-treated liver cells. Another study has reported that EGCG can increase the autophagic level by increasing lysosomal acidification and stimulating autophagic flux in liver cells and the mouse liver [57]. Our data showed that EGCG could increase autophagy in OA-treated liver cells, indicating that autophagic activation can serve as a potential therapeutic target for NAFLD.

It has been shown that low concentrations of intracellular ROS are necessary for many physiological roles including signal transduction and cell proliferation. Nevertheless, ROS overproduction can induce oxidative stress and cellular redox imbalance, thus ultimately affecting many cellular functions [41, 58]. Our data indicated that OA increased ROS levels and decreased the activities of GSH-Px, SOD, and CAT, which were consistent with the results of a previous study [59]. The effects were significantly reversed by the treatment with EGCG. In mammalian cells, three major types of MAP kinases are present: ERK, p38, and JNK, which are associated with EGCG interaction in the MAPK pathway [60, 61]. MAPK cascades play key roles in the progression of NAFLD, and elevation of ROS activates the MAPK pathway [38, 41, 62]. It has been revealed that intraperitoneal administration of EGCG (5 mg/kg) for 14 days inhibits phosphorylation of ERK, JNK, and p38 in animals with artificial unilateral ureteral obstruction [63]. Another study indicates that MAPK and hypoxia-inducible factor-1*α* are decreased after the treatment with EGCG, suggesting that EGCG could suppress MAPK-related oxidative stress [64]. A recent study has shown that EGCG can increase the expression levels of antioxidant enzymes, reverse the increase of ROS production, and regulate mitochondrial-involved autophagy [65]. Furthermore, EGCG could prevent *α*TC1-6 cells from H_2_O_2_-induced ROS production via the activation of Akt signaling and suppression of the p38 and JNK pathway [66]. In this study, OA upregulated the expression levels of p-JNK and p-p38 but downregulated the levels of p-ERK1/2. However, administration of EGCG remarkably reversed the levels of the proteins. Furthermore, the ROS/MAPK pathway is an important signaling cascade which can mediate the processes of apoptosis and autophagy in mammalian cells [67, 68]. In sum, the data suggest that EGCG can reduce apoptosis and induce autophagy possibly through the ROS/MAPK pathway in OA-treated liver cells.

In this study, a mouse model of HFD-induced NAFLD was used to imitate unhealthy dietary habits. Our results suggested that HFD feeding induced obvious increases in body weight, liver weight, white fat weight, and brown fat weight, as well as the levels of TC, TG, ALT, and AST in the plasma of mice, indicating the successful establishment of the NAFLD model. It has been reported that NAFLD patients tend to have higher TC and TG levels [69]. EGCG markedly decreased the levels of TG and TC in both the plasma and the liver. Furthermore, ALT and AST are important indicators of liver damage in NAFLD [70]. Administration of EGCG significantly alleviated liver damage in HFD-fed mice by reducing ALT and AST levels. The flux of NEFA can be delivered to hepatocytes for TG synthesis, resulting in the development of NAFLD [71]. Treatment with EGCG decreased the level of NEFA in the liver of HFD-fed mice. Moreover, EGCG can increase the proliferation and autophagy but decrease the apoptosis in the liver of HFD-fed mice. The data together indicate that EGCG might alleviate HFD-induced NAFLD by inhibiting apoptosis and promoting autophagy.

In summary, our data suggest that EGCG can alleviate HFD-induced NAFLD through inhibition of apoptosis and promotion of autophagy possibly via the ROS/MAPK pathway. EGCG could be developed as an effective agent for the treatment of NAFLD.

## Data Availability

All data generated or analyzed in this study are included in this published article.

## Abbreviations

EGCG: Epigallocatechin-3-gallate
NAFLD: Nonalcoholic fatty liver disease
HFD: High-fat diet
OA: Oleic acid
DMEM: Dulbecco's modified Eagle's medium
FBS: Fetal bovine serum
BSA: Bovine serum albumin
ORO: Oil red O
EdU: 5-Ethynyl-2′-deoxyuridine
CCK-8: Cell counting kit-8
PI: Propidium iodide
GFP: Green fluorescent protein
RFP: Red fluorescent protein
MAP1LC3/LC3: Microtubule-associated protein 1 light chain 3
MDC: Monodansylcadaverine
ROS: Reactive oxygen species
SOD: Superoxide dismutase
CAT: Catalase
GSH-Px: Glutathione peroxidase
CDK: Cyclin-dependent kinase
ERK1/2: Extracellular signal-regulated protein kinase 1/2
p: Phospho
JNK: Jun N-terminal kinase
CST: Cell Signaling Technology
Bcl-2: B-cell lymphoma-2
Bax: Bcl-2-associated X protein
Bcl-xl: B-cell lymphoma-extra large
Bad: Bcl-xl/Bcl-2-associated death promoter
PARP: Poly-ADP-ribose polymerase
LFD: Low-fat diet
TG: Triglyceride
TC: Total cholesterol
ALT: Alanine aminotransferase
AST: Aspartate aminotransferase
NEFA: Nonesterified fatty acid
HE: Hematoxylin and eosin
IHC: Immunohistochemistry
MAPK: Mitogen-activated protein kinase.
